# Lasso Peptide Microcin J25 Effectively Enhances Gut Barrier Function and Modulates Inflammatory Response in an Enterotoxigenic *Escherichia coli*-Challenged Mouse Model

**DOI:** 10.3390/ijms21186500

**Published:** 2020-09-05

**Authors:** Xiuliang Ding, Haitao Yu, Shiyan Qiao

**Affiliations:** 1 State Key Laboratory of Animal Nutrition, Ministry of Agriculture Feed Industry Center, China Agricultural University, Beijing 100193, China; sddingxl@aliyun.com (X.D.); 15600660793@163.com (H.Y.); 2 Beijing Biofeed Additives Key Laboratory, Beijing 100193, China

**Keywords:** enterotoxigenic *Escherichia coli*, microcin J25, intestinal epithelial barrier, intestinal inflammation, mice, pathogen, mitogen-activated protein kinase, nuclear factor κB

## Abstract

Bacterial resistance leads to severe public health and safety issues worldwide. Alternatives to antibiotics are currently needed. A promising lasso peptide, microcin J25 (MccJ25), is considered to be the best potential substitute for antibiotics to treat pathogen infection, including enterotoxigenic *Escherichia coli* (ETEC). This study evaluated the efficacy of MccJ25 in the prevention of ETEC infection. Forty-five female BALB/c mice of clean grade (aged seven weeks, approximately 16.15 g) were randomly divided into three experimental groups as follows: (i) control group (uninfected); (ii) ETEC infection group; (iii) MccJ25 + ETEC group. Fifteen mice per group in five cages, three mice/cage. MccJ25 conferred effective protection against ETEC-induced body weight loss, decrease in rectal temperature and increase in diarrhea scores in mice. Moreover, in ETEC-challenged mice model, MccJ25 significantly improved intestinal morphology, decreased intestinal histopathological scores and attenuated intestinal inflammation by decreasing proinflammatory cytokines and intestinal permeability, including reducing serum diamine oxidase and D-lactate levels. MccJ25 enhanced epithelial barrier function by increasing occludin expression in the colon and claudin-1 expression in the jejunum, ultimately improving intestinal health of host. MccJ25 was further found to alleviate gut inflammatory responses by decreasing inflammatory cytokine production and expression via the activation of the mitogen-activated protein kinase and nuclear factor κB signaling pathways. Taken together, the results indicated that MccJ25 protects against ETEC-induced intestinal injury and intestinal inflammatory responses, suggesting the potential application of MccJ25 as an excellent antimicrobial or anti-inflammation agent against pathogen infections.

## 1. Introduction

*Escherichia coli* (*E. coli*)-caused disease is the most widespread and common type of human or swine intestinal infectious disease in the world [1,2]. Notably, enterotoxigenic *E. coli* (ETEC) is a critical pathogen leading to diarrhea disease, intestinal microecological disorders, enteritis, host intestinal barrier damage and death in young animals and human [3,4,5,6]. Although the ETEC-caused mortality rate in diarrheal diseases has been declining in recent years, ETEC infection still has a significantly high morbidity and mortality worldwide. In addition, ETEC infectious diarrheal disease can cause a high degree of sequelae, leading to repeated intestinal infections [7,8,9]. Given this, the development of anti-infective drugs against ETEC remains a high priority for livestock farming under antiresistance and limited antibiotic use conditions.

To date, a large number of antibiotics are still widely used to prevent and cure enterotoxin-producing *E. coli* infectious diarrhea disease [10,11]. Although antibiotics have excellent bactericidal and anti-inflammatory effects, they can also damage intestinal barrier function, aggravate the intestinal inflammatory response and destroy intestinal homeostasis, eventually leading to host death [12,13,14]. Many studies have shown that direct inhibition or killing of pathogenic microorganisms and regulation of immune function of the host are two ways for antimicrobial peptides (AMPs) to play anti-infection roles [15,16]. With the deep understanding of the structure, function and anti-infection mechanisms of AMPs, clinical research and application of AMPs are being greatly promoted. AMPs have been considered as the most promising potential alternatives to traditional antibiotics [17,18]

Because of the strong antibacterial activity and immune function of microcins, they have been widely used in research [19,20,21]. Microcin J25 (MccJ25) is one of the outstanding members of the microcin family, which is isolated from *E. coli* AY25, characterized by 21 amino acid residues, plasmid coding and ribosome synthesis [22,23,24]. Because of MccJ25′s high antibacterial activity and stable structural characteristics, it attracts the research interest of many researchers [25,26,27]. Furthermore, natural MccJ25 is ribosomally coded, it can be used as a polypeptide bioengineering template to biosynthesize through genetic engineering methods applied in clinical practice [28]. Importantly, recombinant AMPs have been the most commonly used method to produce therapeutic proteins in recent years [29,30,31,32].

In our previous study, the natural MccJ25 gene cluster was transformed by a genetic engineering method, and the high-performance expression vector pMJ25 and high-performance liquid phase purification detection method were established [33,34]. Then, the products with high biologic activity and high purity were obtained [33]. In vitro studies have shown that MccJ25 has strong anti-ETEC activity and that pretreated with MccJ25 protected intestinal porcine epithelial cell J2 (IPEC-J2) cells to prevent ETEC-induced intestinal injury and to alleviate inflammation [34]. However, in vitro approaches have many limitations and require more detailed animal models (e.g., mice or pigs) to evaluate the effects of MccJ25 effects on ETEC-induced intestinal protective barrier dysfunction and the alleviation of inflammatory responses.

## 2. Results

### 2.1. Clinical Symptoms and Intestinal Inflammation

**Figure 1:**
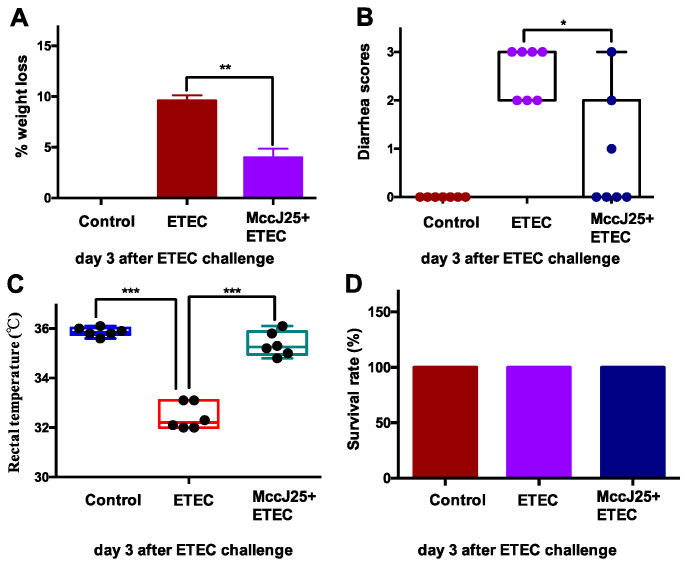
Microcin J25 (MccJ25) significantly improved enterotoxigenic *Escherichia coli* (ETEC)-caused clinical symptoms in mice. (**A**) Body weight loss, (**B**) diarrhea scores, (**C**) rectal temperature and (**D**) survival rate. Data (**A**,**C** and **D**) presented as means ± standard error of the mean (SEM) from seven biologic replicates. Data (**B**) presented as means ± standard error of the mean (SEM) from six biologic replicates. * *p* < 0.05, ** *p* < 0.01, *** *p* < 0.001.

Mice were treated with MccJ25 from Day 1 to Day 7, followed by oral administration of ETEC K88 (5 × 10^10^ Colony forming unit [CFU]/mL]). Results showed that ETEC-infected mice exhibited significantly increased diarrhea scores (Figure 1A), body weight (BW) loss (Figure 1B) and decreased rectal temperature (Figure 1C) compared to the control group (*p* < 0.05). However, administering MccJ25 to mice prior to ETEC infection significantly attenuated the clinical diarrheic clinical symptoms of ETEC-treated mice, including BW loss and diarrhea scores (*p* < 0.05). Additionally, no significant difference in survival rate was observed among these treatments (Figure 1D).

**Figure 2:**
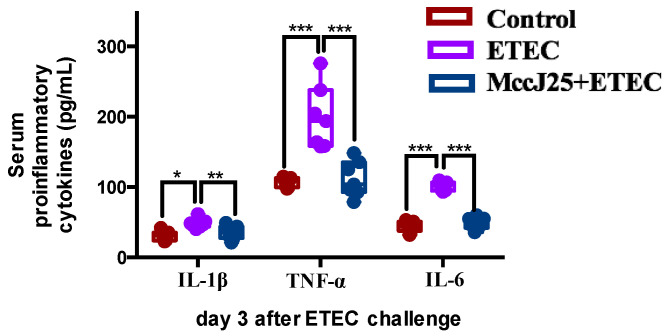
MccJ25 significantly decreased ETEC-induced increases in inflammatory responses. Data presented as means ± SEM from seven biologic replicates. * *p* < 0.05, ** *p* < 0.01, *** *p* < 0.001.

To evaluate the alleviation of intestinal inflammation by pretreated with MccJ25 in mice with ETEC-induced intestinal inflammation, the production of typical inflammatory cytokines, including tumor necrosis factor-α (TNF-α), Interleukin-6 (IL-6), and IL-1β, was evaluated in serum (Figure 2). Compared to the control treatment, ETEC-challenged mice had greater levels of TNF-α, IL-6 and IL-1β in serum (*p* < 0.05). However, oral administration of MccJ25 to mice prior to ETEC infection significantly decreased TNF-α, IL-6 and IL-1β levels compared to the ETEC-treated group (*p* < 0.01).

### 2.2. Pretreated with MccJ25 Inhibited ETEC Colonization

**Figure 3:**
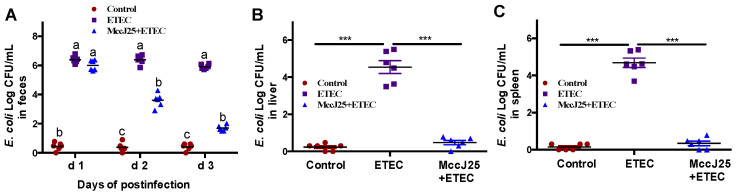
Following oral administration of mice with MccJ25 and subsequent gavage administration of ETEC to examine MccJ25 against pathogens. Bacteria loads in (**A**) fresh feces, (**B**) liver and (**C**) spleen homogenates are shown. Data presented as means ± SEM from 6 biologic replicates. Different superscript lowercase letters (a,b,c) within each group (d1, d2, d3, respectively) indicate significant differences (*p* < 0.05). ^a^: the difference is not significant (*p* > 0.05); ^b^: significant difference (*p* < 0.05); ^c^: remarkable significant difference (*p* < 0.01. *** *p* < 0.001).

Based on these results, we investigated whether pretreated with MccJ25 would protect mice against the expansion of Enterobacteriaceae. Compared to the control treatment group, oral administration of ETEC to mice for 3 d remarkably increased the ETEC counts in feces, liver and spleen in mice (*p* < 0.01). Administering MccJ25 to mice prior to ETEC infection substantially reduced ETEC K88 gut colonization (Figure 3A). Moreover, MccJ25 effectively attenuated the ETEC K88-induced increase in bacterial transfer to the liver and spleen 3 d after the first infection (Figure 3B,C).

### 2.3. Pretreated with MccJ25 Improved Tissue Morphology and Decreased Permeability

**Figure 4:**
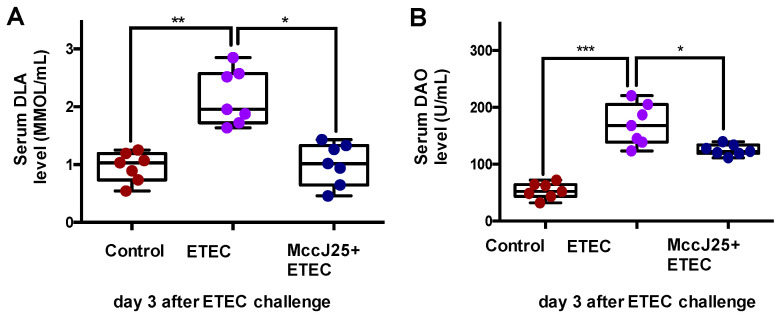
MccJ25 significantly inhibited ETEC-induced intestinal permeability increases. (**A**) DLA levels in serum and (**B**) DAO level in serum. Data presented as means ± SEM from seven biologic replicates. * *p* < 0.05, ** *p* < 0.01, *** *p* < 0.001.

ETEC-infected mice showed significantly increased serum D-lactate (DLA) (Figure 4A) and diamine oxidase (DAO) (Figure 4B) levels compared with the control treatment group (*p* < 0.05). Oral administration of the MccJ25 before ETEC challenge for 7 d markedly reduced DAO (*p* < 0.05) and DLA (*p* < 0.001) levels compared to those of the ETEC-challenged group, thereby decreasing intestinal permeability (Figure 4).

**Figure 5:**
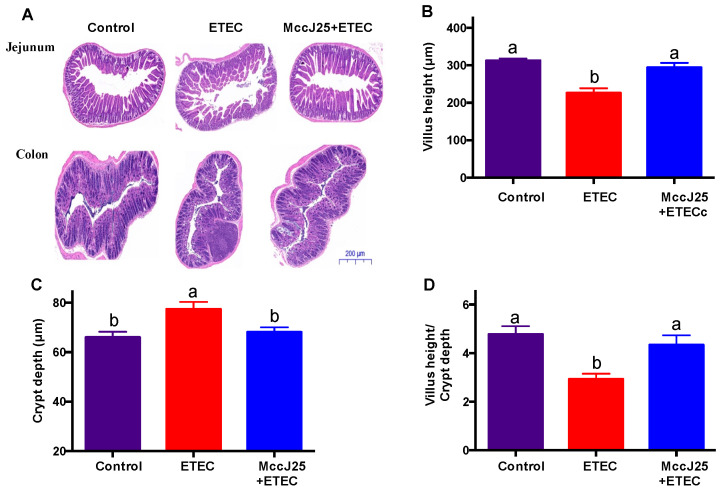

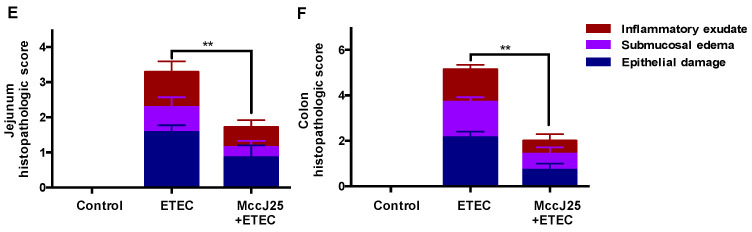
Effects of MccJ25 on the intestinal mucosal morphology and pathologic score. Jejunum and colon sections were used to analyze (**A**) mucosal morphology. (**B**) Villus height, (**C**) crypt depth, (**D**) ratio of the villus length and (**E**) crypt depth and histopathology were determined in (**E**) jejunum and (**F**) colon. Results given as means ± SEM, *n* = 6. Different superscript lowercase letters within each group indicate significant difference (*p* < 0.05). ** *p* < 0.01. ^a^: the difference is not significant (*p* > 0.05); ^b^: significant difference (*p* < 0.05).

Villus morphology and histopathology of the jejunum and colon were assayed (Figure 5). As shown in Figure 5A, the morphology of the villi of the jejunum and colon in the control mice indicated that intestinal epithelial cells were arranged in an orderly fashion and had intact mucus layers. However, compared with the control, ETEC infection resulted in damage to mucosal morphology by increasing histopathology scores (Figure 5A,E,F) and induced lower villous height (*V*), lower *V/C* and higher crypt depth (*C*) in the jejunum compared to the control group (Figure 5B–D) (*p* < 0.05). Compared to ETE-infected mice, pretreated with MccJ25 significantly increased *V* and *V/C* and decreased *C* in the jejunum (*p* < 0.05). Furthermore, MccJ25 pretreatment inhibited ETEC-caused mucosal morphology damage in the jejunum and colon, with slight inflammatory morphology segment impairment persisting in the intestine (Figure 5E,F). In summary, the findings indicate that pretreated with MccJ25 relieved the intestinal inflammation induced by ETEC.

### 2.4. Pretreated with MccJ25 Inhibited ETEC Second Infection

**Figure 6:**
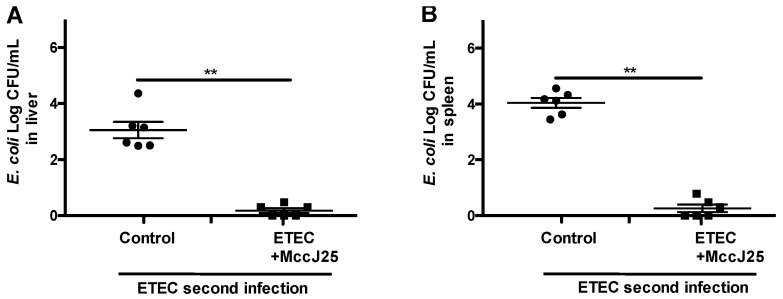
MccJ25 effectively reduced dissemination of bacteria to organs during second infection. Six mice per group were randomly selected from the control and MccJ25 plus ETEC groups were orally challenged with a second ETEC infection. The liver (**A**) and spleen (**B**) tissues were collected for colony counting. All data are expressed as means ± SEM (*n* = 6). Differences were determined via *t*-test. ** *p* < 0.01.

We found that after the second infection, ETEC K88 transfer to the liver (Figure 6A) and spleen (Figure 6B) were significantly decreased in mice treated with MccJ2 (*p* < 0.01). These data indicate that MccJ25-treated mice had improved defense responses against a second ETEC K88 infection.

### 2.5. Pretreated with MccJ25 Improved Intestinal Epithelial Barrier Function

**Figure 7:**
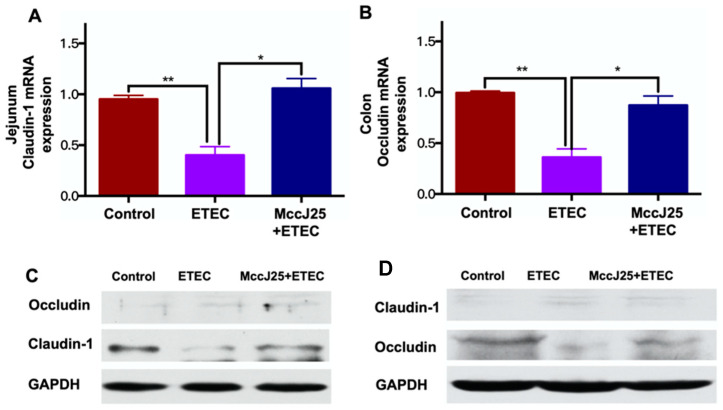
MccJ25 significantly decreased ETEC-induced intestinal epithelial barrier dysfunction. (**A**) *claudin-1* mRNA expression in the jejunum and (**B**) *occludin* mRNA expression in the colon. Data presented as means ± SEM from six biologic replicates. * *p* < 0.05, ** *p* < 0.01. (**C**) Jejunal claudin-1 protein expression in the jejunum and (**D**) colonic occludin protein expression. Data presented as means ± SEM from 3 biologic replicates.

Based on the reinfection results, we further examined the improved intestinal barrier function of mice treated with MccJ25. We further evaluated the impacts of the MccJ25 on gut barrier function in the jejunum and colon by determining tight junction protein (TJP) expression (Figure 7). First, we observed that ETEC challenge sharply decreased mRNA expression of classical TJP *claudin-1* in the jejunum (Figure 7A) and mRNA expression of *occludin* in the colon (Figure 7B) (*p* < 0.05). However, oral MccJ25 effectively inhibited ETEC-induced decrease in *claudin-1* and *occludin* gene expression (*p* < 0.05). Interestingly, no significant difference was observed in terms of *occludin* mRNA expression in the jejunum or *claudin-1* mRNA expression in the colon (data not shown).

To verify the mRNA results, western blot was applied to further validate protein expression of claudin-1 and occludin in the jejunum and colon, respectively. Consistent with the gene expression results, the western blot results showed that ETEC infection decreased expression of claudin-1 protein in the jejunum (Figure 7C) and occludin protein level in the colon (Figure 7D) compared to that of the control group (*p* < 0.05). Compared with the ETEC-infected mice group, oral administration of MccJ25 prior to ETEC challenge significantly increased claudin-1 and occludin protein expression in the jejunum and the colon, respectively (Figure 7C,D) (*p* < 0.05). Notably, no significant difference was observed in terms of occludin protein expression in the jejunum or claudin-1 protein expression in the colon.

### 2.6. Pretreated with MccJ25 Activated Mitogen-Activated Protein Kinase (MAPK) and Nuclear Factor κB (NF-κB) Signaling Pathways to Regulate Inflammation

**Figure 8:**
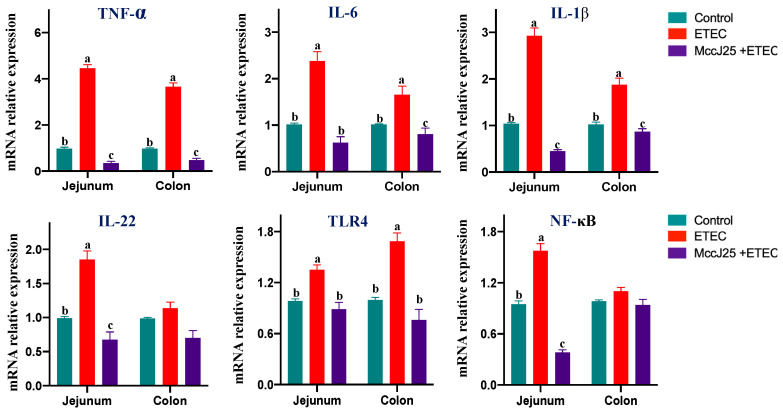
mRNA relative expression analysis of proinflammatory factors in the intestine in ETEC-infected mice. Results expressed as means ± SEM of 3 independent experiments, *n* = 6. Different superscript lowercase letters within each group indicate significant difference (*p* < 0.05). ^a^: the difference is not significant (*p* > 0.05); ^b^: significant difference (*p* < 0.05); ^c^: remarkable significant difference (*p* < 0.01).

To further assess the capacity of MccJ25 to inhibit ETEC-induced intestinal inflammation, mRNA gene expression of pro-inflammatory cytokines in the jejunum and colon were determined using quantitative real-time PCR (qRT-PCR) method. The mRNA results showed that ETEC infection significantly increased the gene expression of pro-inflammatory cytokines *TNF-α*, *IL-6* and *IL-1β*, *IL-22*, *TLR4* and *NF-κB* in the jejunum compared to that of the control group (*p* < 0.05). In the colon, ETEC infection significantly increased the gene expression of pro-inflammatory cytokines *TNF-α*, *IL-6* and *IL-1β* and *TLR4* compared to the control group (Figure 8) (*p* < 0.05). Compared with the ETEC-infected group, oral administration of MccJ25 before ETEC infection significantly decreased gene expression of *TNF-α*, *IL-6* and *IL-1β*, *IL-22*, *TLR4* and *NF-κB* in the jejunum (*p* < 0.05). In the colon, pretreated with MccJ25 significantly decreased *TNF-α*, *IL-6* and *IL-1β* and *TLR4* gene expression (Figure 8) (*p* < 0.05). Notably, for *IL-22*, *TLR4*, *IL-6* and *NF-κB*, no significant differences were observed between control group and pretreated with MccJ25 group in the colon.

**Figure 9:**
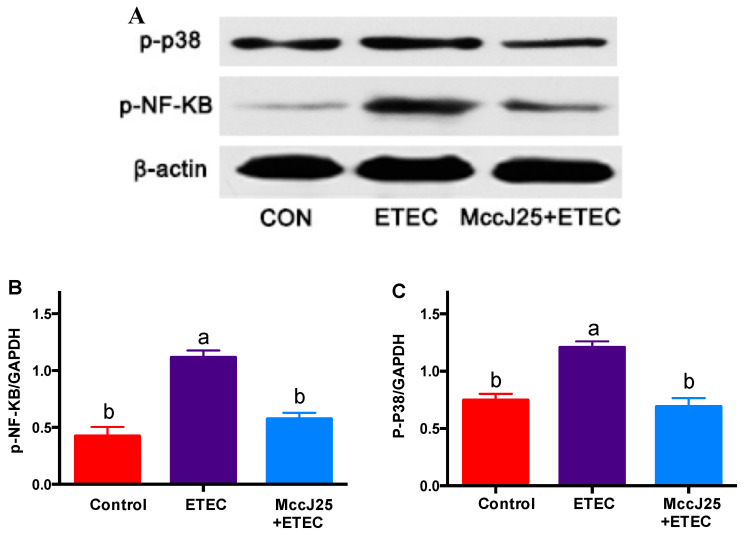
Analysis of MAPK and NF-κB pathway activation in mice by western blotting. (**A**) Western blotting represent image of p-P38 and phosphorylated NF-κB in the jejunum; (**B**) relative p-P38 protein abundance; (**C**) phosphorylated NF-κB. Results given as means ± SEM of 3 independent experiments, *n* = 3. Different superscript lowercase letters within each group indicate significant difference (*p* < 0.05). ^a^: the difference is not significant (*p* > 0.05); ^b^: significant difference (*p* < 0.05).

Furthermore, ETEC-infected mice showed significantly increased abundance of phosphorylated NF-κB (Figure 9A,B) and P38 (Figure 9A,C) protein (*p* < 0.05) compared with all treatment groups. However, compared to the ETEC-treated mice group, pretreated with MccJ25 significantly decreased (*p* < 0.05) the phosphorylated NF-κB and P38 protein abundance in the jejunum. Importantly, there was no significant difference between pretreated with MccJ25 and control groups (*p* > 0.05). These findings indicated that NF-κB and MAPK pathways were activated.

## 3. Discussion

In the current study, we found that the pretreated with mice with MccJ25 as prophylactic drug significantly decreased ETEC counts and effectively attenuated ETEC K88-induced bacterial transfer to the liver and spleen. It was also effective in preventing BW loss, intestinal injury and an increase in the diarrhea score by improving intestinal epithelial barrier function and reducing the inflammatory response. After the second infection, MccJ25-treated mouse still decreased ETEC K88 number, and a significant difference was observed between the MccJ25-treated and untreated mice.

Chronic intestinal inflammatory disorders of the gastrointestinal tract, including inflammatory bowel disease (IBD), are related with increased pro-inflammatory cytokine secretion in the intestines. Intestinal inflammation is a defensive response against pathogen infections and damage in microbiological toxins. Clinical symptoms of IBD include abdominal pain, diarrhea, rectal bleeding, weight loss, malnutrition and fever [35,36,37,38,39]. Diarrheal disease caused by ETEC results in significant worldwide morbidity and mortality in infants and young animals, and it is the leading cause of traveler’s diarrhea [3,40]. Additionally, ETEC-induced inflammation, intestinal epithelial barrier injury and imbalance of gut microbiota, its infection-associated intestinal diseases and even human diseases, such as IBD [41]. Given the strong antimicrobial activity of MccJ25 against ETEC and reduced ETEC adhesion in IPEC-J2 cells [34], we investigated whether MccJ25 could be used to prophylactically treat an active enterobacterial infection in mice prior to infected with ETEC. It is a common appearance that pathogen infection can cause negative impacts on clinical symptoms in host, including BW reduction, diarrhea and death [13,42,43,44,45]. Thus, clinical symptoms are important and obvious parameters that determine the protective ability of antimicrobials in clinical use. In the present study, mice pretreated with MccJ25 was effective in preventing BW loss and rectal temperature decreases, as well as increasing the diarrhea score, thereby improving the health status of mice. Consistent with our present study, a large number of studies indicated that AMPs and microcins improve disease caused by pathogen and enhance the health status of the host [13,19,20,22,27,42,43,44,45].

Moreover, pathogenic bacteria infection is related to IBD [38,39]. Bacterial infection also disrupts the gut microecology and barrier function [46,47]. When the intestinal epithelial barrier is damaged, the gut microbiome is at greater risk of inflammation by increasing the proinflammatory cytokines and infection. To date, the morbidity and mortality caused by infection with enterotoxigenic bacteria, such as *E. coli*, *S. aureus* and *P. aeruginosa*, are largely related to the elicited inflammatory response [42,43,44,45]. AMPs are promising antimicrobial agents because of their strong antimicrobial or bactericidal activities against microorganisms and modulation of inflammatory responses. The AMP MccJ25 was investigated and evaluated largely due to its excellent antimicrobial or bactericidal potential, low toxicity risk in vitro and in vivo and significant improvement in intestinal health of host [33,34]. In this study, we consistently found that pretreated with MccJ25 significantly decreased ETEC counts in the intestines and organs of ETEC-challenged mice.

When exogenous factors such as pathogenic microorganisms, toxins, LPS and other toxic substances enter the intestinal submucosa of the host, intestinal mucosal immune system disorders, hypersensitivity reactions, intestinal IBD and even host death are often induced. One of the main characteristics of long-term or chronic inflammatory responses is the change of secretion and expression of inflammatory factors in the intestine [48,49,50,51,52]. Additionally, pathogen infections cause increases in intestinal permeability, damage intestinal morphology and lead to pathology. For example, a large number of studies have shown that ETEC infection induced IBD in the host, which is accompanied by intestinal mucosal damage, DAO and DLA increases in serum [53,54,55,56], production of a large number of inflammatory factors and aggravation of intestinal epithelial barrier dysfunction, including decrease of Zonula occludes, claudins and occludin [43,44,45].

In this study, the mice pretreated with MccJ25 effectively inhibited ETEC-induced intestinal injury, inflammatory responses and intestinal permeability increased by enhancing intestinal epithelial barrier function. Given the impact of MccJ25 on the function of the intestinal epithelial barrier and microbial community, we investigated the resistance of MccJ25-treated mice to a second ETEC K88 infection. After the second infection, MccJ25-treated mice still showed a decreased ETEC K88 number, and a significant difference was found between the biosynthetic MccJ25-treated and untreated mice. Given the positive effects of MccJ25 on clinical symptoms, intestinal inflammation, the intestinal barrier and microbiota composition after ETEC infection, we can effectively confirm the potential of the MccJ25 to severe as a prophylactic substitute in various industrial domains, including food, humans and animals, especially, in preventing pathogenic bacteria infection-associated intestinal diseases and even human diseases, such as IBD.

NF-κB and MAPK pathways play key roles in intestinal inflammation modulation [57,58,59,60]. A large number of studies have shown that NF-κB and MAPK signaling molecules play roles in proinflammatory cytokine expression [42,61,62]. In this study, our findings indicated that MccJ25 significantly inhibited ETEC-caused expression of inflammatory cytokines, probably by downregulating the MAPK and NF-κB pathways in the jejunum. These results are consistent with other AMPs reducing pathogen-induced proinflammatory factor secretion and expression in the intestine by affecting MAPK and NF-κB pathway in vitro and in vivo [34,63,64,65], suggesting that MccJ25 as a prophylactic drug could be useful to lower inflammation in inflammatory/autoimmune disease of the gut, such as IBD.

## 4. Materials and Methods

### 4.1. Production of MccJ25

Biosynthesis of MccJ25 in our laboratory was performed using a previously constructed high efficiency expression vector pMJ25 and purification (purity > 99.96%) by reverse high-performance liquid chromatograph was described in Yu et al. [33]. Molecular weight and amino acid of MccJ25 were 2107 Da and [GGAGHVPE] YFVGIGTPISFYG, respectively, determined by LC–MS/MS on an ultrahigh-performance LC system connected to a high-resolution ESI-Q-TOF mass spectrometer (Micromass Ltd., Manchester, UK). After incubation, the recombinant bacteria cell supernatants were harvested and freeze-dried using a SpeedVac (Thermo Fisher Scientific, Rockford, IL, USA) to obtain a lyophilized powder and stored at 4 °C. Endotoxin content of MccJ25 was less than 0.005 EU/mg.

### 4.2. Preparation of ETEC Strain

F4^+^ ETEC was selected and utilized in the present study (serotype O149:K88ac). The indicator bacterium was obtained from the China Institute of Veterinary Drug Control (Beijing, China) and stored at our laboratory. First, the ETEC strain was seeded, identified and purified on MacConkey agar (Beijing AoBoXing Biotechnology Co., Ltd., Beijing, China) to obtain a single pure colony. Then, a single colony was inoculated into Luria–Bertani (LB) broth (Beijing AoBoXing Biotechnology Co., Ltd., Beijing, China) or incubated overnight at 37 °C, 200 rpm/min). Then, 100 μL overnight cultures were transferred to 10 mL fresh LB medium and incubated at 37 °C and centrifuged at 200 rpm/min for 2 h 10 min. After this, 1 mL cultures were detected at the absorbance (OD) value of 600-nm wavelength. When OD_600_ = 1.0 (corresponding to bacterial concentration of approximately 5 × 10^8^ CFU/mL) the bacteria were collected and used.

### 4.3. Experimental Animals

All experiments were referred and used to guide the process of the present study were according to the guidelines of the Ethics Review Committee of China Agricultural University Institutional Animal Care and Use Committee (ICS 65.020.30, 5 May 2016) and carried out in compliance with the National research Council’s Guide for the Care and Use of Laboratory Animals.

Six-week-old female clean-grade BALB/c mice with initial BW of 15.12 ± 0.53 g and were purchased from Beijing Huafukang Biologic Company. Mice were kept and acclimatized under the new sterilized environment for 3 days prior to the beginning of the infection experiment. Mice were individually housed in the same temperature- and humidity-controlled room on a 12-h light/dark cycle with ad libitum access to feed and water during the experimental period. Feces were collected and plated on the ChromAgar and MacConkey agar (Beijing AoBoXing Biotechnology Co., Ltd., Beijing, China), demonstrating that culturable enterobacteria and ETEC were absent from the mice.

### 4.4. Experimental Design

**Figure 10:**
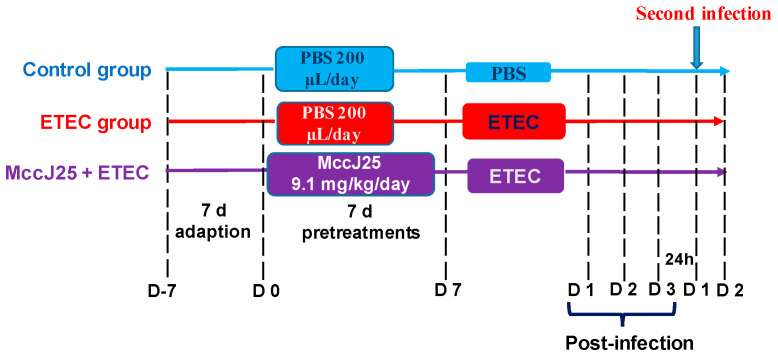
Scheme of animal treatments.

As shown in Figure 10, after a 7-d acclimatization period, 45 female BALB/c mice of clean grade (aged 7 weeks, approximately 16.15 g) were randomly divided into three experimental groups (15 mice per group in five cages, three mice/cage). Prior to infection, the control and ETEC group received 0.3 mL of sterile phosphate buffer saline (PBS) (Beijing Boruichangyuan Technology Co., Ltd., Beijing, China) by gavage, whereas the MccJ25 plus ETEC groups received 9.1 mg/kg BW of MccJ25 in a total volume of 0.3 mL of sterile PBS by gavage for 7 d. The concentration of MccJ25 was selected based on our previous study [33]. Subsequently, ETEC and MccJ25 plus ETEC groups received 0.5 mL of sterile PBS containing 5 × 10^11^ CFU/mL of ETEC K88 for 3 d.

### 4.5. Clinical Symptoms and Samples Collection

After ETEC infection, BW, diarrhea scores and survival rates were determined. Fresh stool samples were collected daily for microbial analysis. For diarrhea scores assessments, the clinical status of the mice was monitored daily with a scoring system, as described previously [61]. After 3 d, mice were euthanized, and the fresh livers and spleens were harvested for microbial counts. Intestinal tissues (jejunum and colon) were collected, immediately stored in 4% paraformaldehyde (Beijing Boruichangyuan Technology Co., Ltd., Beijing, China) and snap-frozen in liquid nitrogen and stored in −80 °C for subsequent analysis. Blood was collected, and serum was obtained after centrifugation (Model Biofuge22R, Heraeus, Hanau, Germany) at 3500× *g* for 15 min at 4 °C and then stored in −80 °C until analysis.

### 4.6. Bacterial Transfer during Second Infection of ETEC

After the ETEC-infected mice were treated as described in the “Experimental design” section above (Figure 10), six mice from the control and MccJ25 plus ETEC groups were randomly selected. After 3 d of treatment, all selected mice were orally rechallenged with 10^9^ CFU/mL of ETEC. After 24 h, all mice were euthanized, and livers and spleens of mice were collected and homogenized in cold PBS. The numbers of bacteria were determined by plating serial dilutions on LB agar plates.

### 4.7. Fecal Microbiota Count

The amount of fecal microbiota compositions in mice was determined and calculated by the dilution counting method. Fresh feces of mice were collected every morning. Then, fecal samples were suspended in sterile PBS and vortex the cultures (0.5 g of each fresh fecal sample was assigned to 4.5 mL of sterile saline). After this, appropriate dilutions of 100-μL homogenates were spread over chromogenic ETEC medium and MacConkey agar plates to count colonies. Some colonies growing on MacConkey agar plates were taken to determine whether they belong to Enterobacteriaceae. Plates were incubated in a 37 °C incubator for 24 h under anaerobic conditions and the results were shown as CFU/g feces. The test was repeated three times.

### 4.8. Proinflammatory Cytokines Detection and Intestinal Permeability Analysis

Levels of anti-inflammatory cytokines IL-1β, IL-6 and TNF-α in the jejunum and colon of mice were determined using enzyme-linked immunosorbent assay (ELISA) kits (Nanjing Jiancheng Bioengineering Institute, Nanjing, China) according to the kit standard procedure. Total jejunal and colonic proteins were lysed with lysis buffer (Huaxingbio Biotechnology, Beijing, China) and the protein ratios in the supernatants was determined using BCA protein (Thermo Fisher Scientific, Rockford, IL, USA) based on the kit instructions.

The serum DLA and DAO of mice were determined to analyze the intestinal permeability using the corresponding ELISA kits (Nanjing Jiancheng Bioengineering Institute, Nanjing, China) and the operation steps were carried out according to the instructions.

### 4.9. Pathologic Score and Tissue Morphology

The jejunal and colonic intestinal segments were fixed with 4% paraformaldehyde (Beijing Boruichangyuan Technology Co., Ltd., Beijing, China) to prepare paraffin sections. After 48 h of fixation, the jejunum and colon were analyzed for tissue morphology and pathologic scores. After slice preparation, hematoxylin–eosin (H&E) staining was performed. Each slice in each group was photographed with a microscope containing an integrated digital imaging analysis system (Olympus BX51, Olympus, Japan) in a visual field ×200 to observe tissue sections and determine intestinal villus morphology. We tried to fill the organization with the whole field of vision and ensure that the background light of each photo was consistent. Image-Pro Plus 6.0 (Media Cybernetics, Bethesda, MD, USA) software was used to measure villus height (μm) and crypt depth (μm). The ratio of villus height to crypt depth (Villus height:Crypt depth) was calculated. Intestinal pathologic changes in the various treatment groups were assessed according to the degree of submucosal edema, inflammatory cell infiltration, epithelial cell necrosis, central chylous tube dilatation and lesion. The lesions were graded according to standard grades: no lesion, 0; mild injury, 1; moderate injury, 2; severe injury, 3.

### 4.10. qRT-PCR

The mRNA expression levels of *claudin-1* in the jejunum, *occludin* in the colon and proinflammatory cytokines in the jejunum and colon were determined using qRT-PCR as previously described with minor modifications [66]. Briefly, total RNA was extracted from the jejunum, ileum, spleen and cells using the TRIzol reagent (Invitrogen, Carlsbad, CA, USA). The quality and quantity of total RNA were determined using gel electrophoresis and a NanoDrop 2000 spectrophotometer (Thermo Fisher Scientific, Wilmington, DE, USA). The first-strand cDNA was synthesized from the extracted RNA (1 μg) using a Prime-Script 1st Strand cDNA synthesis kit (Takara, Otsu, Japan) according to the manufacturer’s instructions. qRT-PCR was performed on a LightCycler (Roche, Basel, Switzerland) with SYBR Green PCR Master Mix (Takara, Otsu, Japan). The relative amounts of mRNAs were normalized against *GAPDH* and analyzed using the 2^−ΔΔCt^ method. The primers of *claudin-1*, *occludin* and proinflammatory cytokines are displayed in Table 1.

### 4.11. Western Blotting

Protein expression levels of claudin-1 and occludin in the jejunum and in the colon, respectively and phosphorylated P38 and phosphorylated NF-κB in the jejunum were determined using Western blotting as previously described with minor modifications [34]. Frozen tissue samples and fresh cells were homogenized in RIPA lysis buffer (Applygen, Beijing, China) containing protease inhibitors (Applygen, Beijing, China). Protein concentrations were determined using a BCA Protein Assay Kit (Thermo Fisher Scientific, Rockford, IL). Samples of 30 μg of protein were electrophoresed on SDS polyacrylamide gels and electrotransferred onto PVDF membranes (Millipore, Billerica, MA, USA). The membranes were blocked with 1 × Tris buffered saline Tween (TBST) containing 5% BSA (Sigma-Aldrich, St Louis, MO, USA) for 2 h at room temperature and then incubated with the corresponding primary antibodies (1:1000 dilutions overnight at 4 °C) for claudin-1 in the jejunum and occludin in the colon (Cell Signaling Technology, Boston, MA, USA). After washing with 1 × TBST (Huaxingbio Biotechnology, Beijing, China), the membranes were incubated with horseradish peroxidase-conjugated goat anti-mouse IgG (Huaxingbio Biotechnology, Beijing, China) for 1 h at room temperature. Chemifluorescence was detected using western blot luminance reagent (Huaxingbio Biotechnology, Beijing, China) using an ImageQuant LAS 4000 mini system (GE Healthcare Biosciences AB, Inc., Stockholm, Sweden) and quantified using a gel imaging system with Image Quant TL (GE Healthcare Life Science, Pittsburgh, PA, USA).

### 4.12. Statistical Analysis

The results are expressed as means ± SEM. Data were analyzed using one-way ANOVA with Prism 6 software (GraphPad Software, Inc., San Diego, CA, USA). Tukey’s post hoc test was used to determine differences between treatments. All data were visualized using Prism 8. *p*-values < 0.05 indicated statistical significance. All experiments were carried out three independently times unless otherwise stated.

## 5. Conclusions

In summary, our findings provide a theoretical basis for the application of MccJ25 as a new feed additive or new veterinary medicine in the breeding industry, as well as provide new means for the realization of feed nonresistance and breeding anti-resistance reduction, improving the production performance of young animals, reducing the mortality of newborns and promoting the development of the livestock industry. The functions and anti-infection mechanisms of MccJ25 are important for the promotion of development and the application of antibiotic substitutes. It can be used to establish effective strategies for the prevention and control of pathogenic microorganism infection in the pig industry and other fields in China, showing great application prospects and potentially meeting the new needs for the development of the national economy in China. Moreover, MccJ25 could be used to treat several intestinal inflammation conditions, such as IBD, by anti-inflammatory function.

## Figures and Tables

**Table 1:** qRT-PCR primer sequences applied in this study.

Genes	Orientation	Sequence (5′-3′)	Size (bp)	NCBI Gene ID
*GAPDH*	Forward	GAGAAACCTGCCAAGTATGATGAC	212	NM_017008.3
	Reverse	TAGCCGTATTCATTGTCATACCAG		
*TNF-α*	Forward	CCACGCTCTTCTGTCTACTG	169	NM_010851.2
	Reverse	ACTTGGTGGTTTGCTACGAC		
*IL-6*	Forward	GAGTCACAGAAGGAGTGGCTAAGGA	106	NM_031168.1
	Reverse	CGCACTAGGTTTGCCGAGTAGATCT		
*IL-1β*	Forward	GGACAGCCTGTTACTACCTGACACATT	239	NM_031512
	Reverse	CCTAGGAAACAGCAATGGTCGGGAC		
*IL-22*	Forward	GACAGGTTCCAGCCCTACAT	166	NM_016971.1
	Reverse	TCGCCTTGATCTCTCCACTC		
*TLR4*	Forward	GTTTGCTCAGGATTCGAGGC	160	AF185285.1
	Reverse	CCGTCGTGTAGTCTGTCTCGTA		
*NF-κB*	Forward	CCTTCCGCAAACTCAGCTTT	173	NM_008689.2
	Reverse	GGACGATGCAATGGACTGTC		
*Claudin-1*	Forward	GCTGGGTTTCATCCTGGCTTCT	110	NM_016674.4
	Reverse	CCTGAGCGGTCACGATGTTGTC		
*Occludin*	Forward	GTGGTAACTTGGAGGCGTCTTC	102	NM_001163647.2
	Reverse	CCGTCGTGTAGTCTGTCTCGTA		

## Abbreviations

MccJ25	microcin J25
AMPs	Antimicrobial peptides
*E. coli*	*Escherichia coli*
ETEC	Enterotoxigenic *E. coli*
TNF-α	Tumor necrosis factor-α
IL	Interleukin
TJP	Tight junction protein
DAO	Diamine oxidase
DLA	D-lactate
CFU	Colony forming unit
HRP	Horse radish peroxidase
V	Villous height
C	Crypt depth
MAPK	Mitogen-activated protein kinase
NF-κB	Nuclear factor κB
qRT-PCR	Quantitative real-time PCR
BW	Body weight
SEM	Standard error of the mean
PBS	Phosphate buffer saline
ELISA	Enzyme-linked immunosorbent assay
LB	Luria–Bertani
IPEC-J2	Porcine epithelial cell J2
IBD	Inflammatory bowel disease
